# Levels of Fibrinogen Variants Are Altered in Severe COVID-19

**DOI:** 10.1055/a-2102-4521

**Published:** 2023-07-13

**Authors:** Judith J. de Vries, Chantal Visser, Maureen van Ommen, Casper Rokx, Els van Nood, Eric C. M. van Gorp, Marco Goeijenbier, Johannes P. C. van den Akker, Henrik Endeman, Dingeman C. Rijken, Marieke J. H. A. Kruip, Miranda Weggeman, Jaap Koopman, Moniek P. M. de Maat

**Affiliations:** 1 Department of Hematology, Erasmus MC, University Medical Center Rotterdam, Rotterdam, The Netherlands; 2 Fibriant BV, Leiden, The Netherlands; 3 Department of Medical Microbiology and Infectious Diseases, Erasmus MC, Erasmus Medical Center Rotterdam, Rotterdam, The Netherlands; 4 Department of Internal Medicine, Erasmus MC, Erasmus Medical Center Rotterdam, Rotterdam, The Netherlands; 5 Department of Viroscience, Erasmus MC, Erasmus Medical Center Rotterdam, Rotterdam, The Netherlands; 6 Department of Adult Intensive Care, Erasmus Medical Center Rotterdam, Rotterdam, The Netherlands

**Keywords:** splicing, COVID-19, fibrinogen, fibrin, inflammation

## Abstract

**Background** Fibrinogen variants as a result of alternative messenger RNA splicing or protein degradation can affect fibrin(ogen) functions. The levels of these variants might be altered during coronavirus disease 2019 (COVID-19), potentially affecting disease severity or the thrombosis risk.

**Aim** To investigate the levels of fibrinogen variants in plasma of patients with COVID-19.

**Methods** In this case-control study, we measured levels of functional fibrinogen using the Clauss assay. Enzyme-linked immunosorbent assays were used to measure antigen levels of total, intact (nondegraded Aα chain), extended Aα chain (α _E_ ), and γˊ fibrinogen in healthy controls, patients with pneumococcal infection in the intensive care unit (ICU), ward patients with COVID-19, and ICU patients with COVID-19 (with and without thrombosis, two time points).

**Results** Healthy controls and ward patients with COVID-19 ( *n* = 10) showed similar fibrinogen (variant) levels. ICU patients with COVID-19 who later did ( *n* = 19) or did not develop thrombosis ( *n* = 18) and ICU patients with pneumococcal infection ( *n* = 6) had higher absolute levels of functional, total, intact, and α _E_ fibrinogen than healthy controls ( *n* = 7). The relative α _E_ fibrinogen levels were higher in ICU patients with COVID-19 than in healthy controls, while relative γˊ fibrinogen levels were lower. After diagnosis of thrombosis, only the functional fibrinogen levels were higher in ICU patients with COVID-19 and thrombosis than in those without, while no differences were observed in the other fibrinogen variants.

**Conclusion** Our results show that severe COVID-19 is associated with increased levels of α _E_ fibrinogen and decreased relative levels of γˊ fibrinogen, which may be a cause or consequence of severe disease, but this is not associated with the development of thrombosis.

## Introduction

Severe acute respiratory syndrome coronavirus 2 (SARS-CoV-2) is the cause of coronavirus disease 2019 (COVID-19), mainly targeting the respiratory tract, leading to coughing, fever, and in severe cases, pneumonia. In these severe cases, an increased incidence of thrombotic complications has been reported. 1 The disease burden and mortality of thrombotic diseases are influenced by the architecture and stability of a thrombus. 2 Upon cleavage of fibrinogen by thrombin, fibrin monomers form. These fibrin monomers start polymerizing, finally forming fibrin fibers that are cross-linked by factor (F)XIII resulting in a stable fibrin network, one of the main components in a thrombus. 3 Fibrinogen is a glycoprotein of 340 kDa produced in the liver and consists of two sets of three different polypeptide chains: Aα, Bβ, and γ. 4 Variation in the fibrinogen molecule occurs due to genetic polymorphisms, alternative messenger RNA (mRNA) processing, proteolytic cleavage, and posttranslational modifications. 5 6 The structure of the fibrin network is affected by these fibrinogen variants.

Proteolytic cleavage of the C-terminus of one or two of the Aα chains leads to low-molecular-weight (LMW, 305 kDa) and low-molecular-weight prime (LMWˊ, 270 kDa) fibrinogen, respectively. 7 The part of the Aα chain removed during this cleavage contains functional domains affecting polymerization and lateral aggregation of protofibrils, thereby influencing the thickness of the fibrin fibers and the fibrin network structure. 8 9 Fibrin fibers formed from LMW fibrinogen are indeed thinner than fibrin fibers formed from high-molecular-weight fibrinogen, 10 resulting in a denser fibrin network. 11 In addition, the C-terminus of the Aα chain contains binding sites for endothelial cells, plasminogen, and factor XIII, thereby also affecting other processes in which fibrinogen or fibrin is involved. 11

Other common variants of fibrinogen occur as a result of alternative mRNA splicing, such as an extension of the Aα chain (α _E_ fibrinogen). α _E_ fibrinogen represents typically 1 to 2% of the total fibrinogen molecules (as measured by quantitative western blot) and is only present as a homodimer of two extended Aα-chains. 12 It is produced upon splicing an extra exon into the Aα-chain mRNA, leading to an additional globular domain at the C-terminus. 12 13 This extension contains a binding site for β2-integrins, possibly enabling leukocytes to bind to fibrinogen. This additional domain also affects fibrin polymerization, resulting in thinner fibers, increased branching, and an increased stiffness of clots prepared from purified α _E_ fibrinogen. 13

The mRNA splice variant γˊ derives from the replacement of the last four amino acids of the γ chain by 20 other amino acids, leading to an extended γ chain. 14 Between 5 and 15% of fibrinogen molecules are heterodimers of γˊ with the normal γ chain (γA/γˊ) and less than 1% are homodimers of γˊ. 15 The variation occurs in the D-region of the fibrinogen molecule, thereby affecting fibrin polymerization, decreasing platelet binding and increasing binding of thrombin and FXIII. 16 17 18 Studies have reported thinner fibers and a more branched network in clots made with γA/γˊ fibrinogen compared to clots prepared from γA/γA fibrinogen. 19 20 21 γˊ fibrinogen levels can vary largely between individuals and are associated with various diseases. 22 23 24 25 26 27

Since fibrinogen variants were previously associated with various thrombotic diseases and an altered fibrin network structure, we hypothesized that these fibrinogen variants would be increased in patients with severe COVID-19 and thrombosis. Therefore, we investigated whether levels of functional fibrinogen, total fibrinogen, intact fibrinogen, γˊ fibrinogen, and α _E_ fibrinogen are altered in COVID-19 and whether this can explain why some patients with COVID-19 develop thrombosis and others do not.

## Methods

### Study Design and Patient Population

This study was a case-control study conducted in the Erasmus Medical Center in Rotterdam, the Netherlands, as part of the Dutch COVID and Thrombosis Coalition. 28 The patients and laboratory measurements are described previously. 29 Briefly, we collected citrated platelet-poor plasma samples between April and December 2020. Samples were collected from patients with COVID-19 admitted to the intensive care unit (ICU) who did and did not develop thrombosis during their stay at the ICU as confirmed by positive or negative computed tomography pulmonary angiograms (performed for all patients with COVID-19) and compression ultrasound of the extremities (only performed if symptoms compatible with venous thrombosis were present). Samples were collected before and after diagnosis of thrombosis or at similar time points in ICU patients without confirmed thrombosis. Additionally, we collected plasma from patients with COVID-19 admitted to general wards who did not have thrombosis, SARS-CoV-2-negative ICU patients with pneumococcal infection, and healthy controls. 30 Study protocols were in accordance with the Declaration of Helsinki and were approved by the Medical Ethics Committee of Erasmus Medical Center (healthy controls: MEC-2004-251; pneumococcal ICU patients: MEC-2017-417; COVID-19 patients: METC-2020-0758). We obtained written informed consent from each healthy control and ICU patient with pneumococcal infection. An opt-out procedure was in place for the patients with COVID-19. Functional fibrinogen levels were measured using the Clauss assay (Thrombin Reagent, Siemens Healthineers, Erlangen, Germany) on the Sysmex CS5100 coagulation analyzer (Siemens Healthcare Diagnostics B.V., Newark, Delaware, United States).

### Fibrinogen Variant ELISAs

We used enzyme-linked immunosorbent assays (ELISAs) based on monoclonal antibodies to measure antigen levels of total, intact, γˊ and α _E_ fibrinogen. First, 96-well MaxiSorp plates (439454, Thermo Fisher Scientific, Waltham, Massachusetts, United States) were coated overnight at 37°C with 120 µL coating antibody in phosphate-buffered saline (PBS). A fibrinogen polyclonal antibody (GaHu/Fbg/7S, Thermo Fisher Scientific) (10 µg/mL) and the G8 monoclonal antibody targeting the C-terminus of the Aα chain (FB-G8-1-2, Quickzyme, Leiden, the Netherlands) (10 µg/mL) were used as coating antibodies for total and intact fibrinogen, respectively. For both ELISAs, reference lines were prepared using purified human fibrinogen (FIB3, Enzyme Research Laboratories, South Bend, Indiana, United States). The 2.G2.H9 antibody (1 µg/mL) (sc-81620, Santa Cruz, Dallas, Texas, United States) 27 and α _E_ antibody (1 µg/mL) (ab247586, Abcam, Cambridge, United Kingdom) were used as coating antibodies for γˊ fibrinogen and α _E_ fibrinogen, respectively. Reference lines were prepared with Peak 2 (P2 FIB, Enzyme Research Laboratories) and rhFib α _E_ (kind gift of Fibriant BV). After incubation of 100 µL diluted plasma (independent triplicates per sample) for 1 hour at 37°C, plates were washed using PBS with 0.05% Tween 20 (524653, Merck Millipore, Burlington, Massachusetts, United States) and incubated with Y18/PO conjugate (FB-Y18-4, Quickzyme) (1:10.000 × ) for 1 hour at 37°C. After thorough washing, each well was incubated with 100 µL 3,3′-5,5′-tetramethylbenzidine (TMB) (TMB Ultra, WD3243711, 34029, Thermo Fisher Scientific). To stop the substrate reaction, 100 µL of 2 M sulfuric acid was added to each well, after which the absorbance was measured at 450 nm using the Multiskan GO Microplate Spectrophotometer (Thermo Fisher Scientific). Results were calculated based on the four-parameter logistic fit using the SkanIt software (Thermo Fisher Scientific). Relative levels of α _E_ and γˊ fibrinogen were calculated as percentage of total fibrinogen measured using the GaHu/Fbg/7S antibody.

### Fibrin Network Characteristics

To study the characteristics of the fibrin network, clots were prepared from the citrated platelet-poor plasma and imaged as described previously. 29 Plasma clot lysis time was measured to investigate the susceptibility of plasma clots to fibrinolysis, as described previously. 29

### Statistical Analysis

Normally distributed data are shown as mean ± standard deviation, not-normally distributed data as median [25th–75th percentile], and categorical data as *n* (%). To test for differences between multiple groups, one-way ANOVA (normally distributed data), Kruskal–Wallis test (not-normally distributed data), or Chi-square test (categorical data) was used with post-hoc Tukey's tests. Changes in variables between the two time points were evaluated using the paired students' *t* -test (normally distributed data) or Wilcoxon signed-rank test (not-normally distributed data). Correlations were assessed using Spearman's rank correlation. We used pairwise deletion in case of missing data. Statistical analyses were performed using IBM SPSS Statistics v25 (IBM, Armonk, New York, United States) and GraphPad Prism version 8.2.1 (GraphPad Software, San Diego, California, United States).

## Results

### Baseline Patient Characteristics

Patient characteristics at the first time point are shown in Table 1 . Of the 19 ICU patients with COVID-19 and confirmed thrombosis, 16 had pulmonary thrombosis, 1 deep venous thrombosis, 1 pulmonary thrombosis in combination with deep venous thrombosis, and 1 jugular vein thrombosis. The diagnosis of thrombosis in the ICU patients with COVID-19 and thrombosis was made after a median of 10 [6–17] days in the ICU. Furthermore, we had plasma samples from 18 ICU patients with COVID-19 without confirmed thrombosis, 10 ward patients with COVID-19 without confirmed thrombosis, 6 ICU patients with pneumococcal infection, and 7 healthy controls. Mean age and sex were comparable, while body mass index was slightly higher in ward and ICU patients with COVID-19 than in healthy controls ( Table 1 ). Results from laboratory measurements can be found in Table 1 .

**Table 1:** Patient characteristics at the first time point

	Healthy controls ( *n* = 7)	Ward patients with COVID-19 ( *n* = 10)	ICU patients with COVID-19 without thrombosis ( *n* = 18)	ICU patients with COVID-19 with thrombosis ( *n* = 19)	ICU patients with pneumococcal infection ( *n* = 6)	*p* -Value
Age (y)	57.0 ± 4.7	60.2 ± 10.6	56.5 ± 15.8	57.8 ± 14.9	61.0 ± 8.3	0.93
Male	2 (29%)	5 (50%)	12 (67%)	13 (68%)	3 (50%)	0.37
Body mass index	23.2 ± 2.1	30.8 ± 6.5	30.9 ± 8.0 a	29.8 ± 4.8	27.4 ± 6.4	0.07
Days since (ICU) admission	–	3 [2–6]	5 [3–8] b	2 [1–6] b	0 [0–0]	**<0.01**
Anticoagulation						**<0.01**
None	7 (100%)	0 (0%)	0 (0%)	0 (0%)	0 (0%)	
Standard prophylaxis	0 (0%)	10 (100%)	6 (33%)	2 (11%)	5 (84%)	
Intermediate prophylaxis	0 (0%)	0 (0%)	10 (56%)	14 (74%)	0 (0%)	
Therapeutic	0 (0%)	0 (0%)	2 (11%)	3 (16%)	1 (17%)	
Anti-Xa (U/mL)	<0.10	0.17 ± 0.12	0.37 ± 0.22 a	0.54 ± 0.28 a b c	0.29 ± 0.25	**<0.01**
Corticosteroids	–	7 (70%)	11 (61%)	10 (53%)	2 (33%)	**0.04**
Mortality	0 (0%)	0 (0%)	1 (6%)	4 (21%)	0 (0%)	0.37
Laboratory measurements						
C-reactive protein (mg/L)	NA	15 [12–45]	91 [68–157] b c	167 [88–240] c	305 [207–349] c	**<0.01**
Interleukin-6 (pg/mL)	NA	NA	59 [20–110]	32 [14–134]	NA	0.74
Procalcitonin (ng/mL)	NA	NA	0.37 [0.24–0.57]	1.26 [0.30–12.48]	NA	0.07
FVIII (U/mL)	0.81 ± 0.27	2.61 ± 1.12 a	3.39 ± 1.20 a	3.07 ± 1.17 a	2.37 ± 1.24	**<0.01**
FXIII (U/mL)	1.32 ± 0.19	1.36 ± 0.24	0.85 ± 0.25 a c	0.95 ± 0.27 a c	0.81 ± 0.58 a c	**<0.01**
D-dimer (mg/L)	0.21 [0.19–0.28]	0.41 [0.26–0.75]	1.02 [0.75–2.07] a	1.35 [0.85–3.26] a c	1.30 [0.98–7.18] a	**<0.01**
Plasminogen activator inhibitor 1 (ng/mL)	<0.3	3.5 [2.7–4.8]	6.5 [4.5–8.0] a	10.2 [4.5–32.3] a c	13.3 [3.3–32.2] a	**<0.01**

Abbreviations: COVID-19, coronavirus disease 2019; FXIII, factor XIII; ICU, intensive care unit; NA, not available.

Note: Mean ± SD, median [25th–75th percentile], or *n* (%) is given. Statistically significant *p* -values are indicated in bold.

a Significantly different from healthy controls.

b Significantly different from ICU patients with pneumococcal infection.

c Significantly different from ward patients with COVID-19.

### Levels of Fibrinogen Variants

**Fig. 1:**
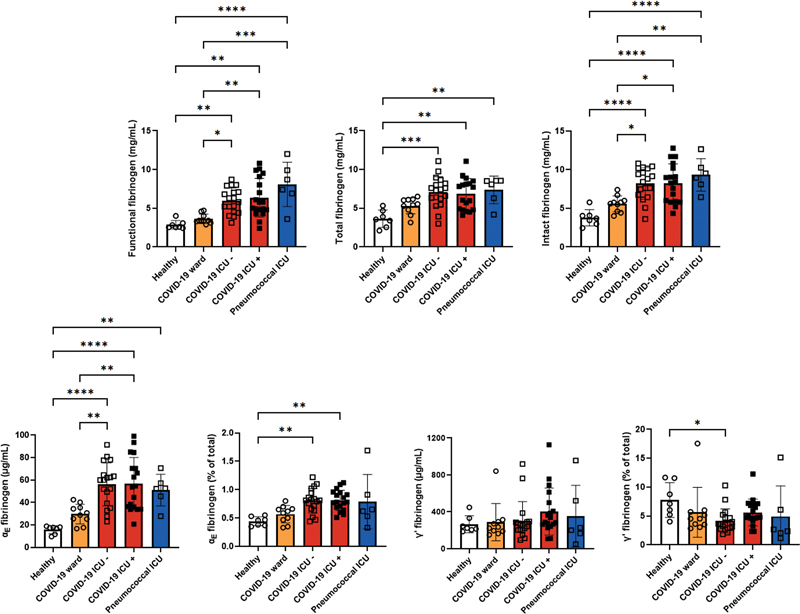
Levels of fibrinogen (variants) in plasma collected from healthy controls, COVID-19 ward patients with COVID-19, pneumococcal ICU patients, ICU patients with COVID-19 without thrombosis (COVID-19 ICU −), and ICU patients with COVID-19 and thrombosis before their diagnosis of thrombosis (COVID-19 ICU +). * *p* < 0.05, ** *p* < 0.01, *** *p* < 0.001, **** *p* < 0.0001. COVID-19, coronavirus disease 2019; ICU, intensive care unit.

First, we analyzed plasma samples from healthy volunteers and from all patients collected at the first available time point after admission to the hospital ( Fig. 1 and Supplementary Table S1 ). Levels of fibrinogen and fibrinogen variants were not significantly different in ward patients with COVID-19 compared to healthy controls. In ICU patients with COVID-19 with and without thrombosis and in ICU patients with pneumococcal infection, we observed significantly higher absolute levels of functional fibrinogen, total fibrinogen, intact fibrinogen, and α _E_ fibrinogen than in healthy controls. Levels of functional fibrinogen, intact fibrinogen, and α _E_ fibrinogen were also significantly higher in all ICU patients than in ward patients with COVID-19. Relative levels of α _E_ fibrinogen were significantly higher in ICU patients with COVID-19 with and without thrombosis than in healthy controls. Finally, the absolute levels of γˊ fibrinogen were not different among the different groups. The relative levels of γˊ fibrinogen showed a trend toward lower levels in patients with COVID-19, which only reached statistical significance in ICU patients with COVID-19 without thrombosis compared to healthy controls. No differences in fibrinogen (variant) levels were observed between ICU patients with COVID-19 who did and did not develop thrombosis.

**Fig. 2:**
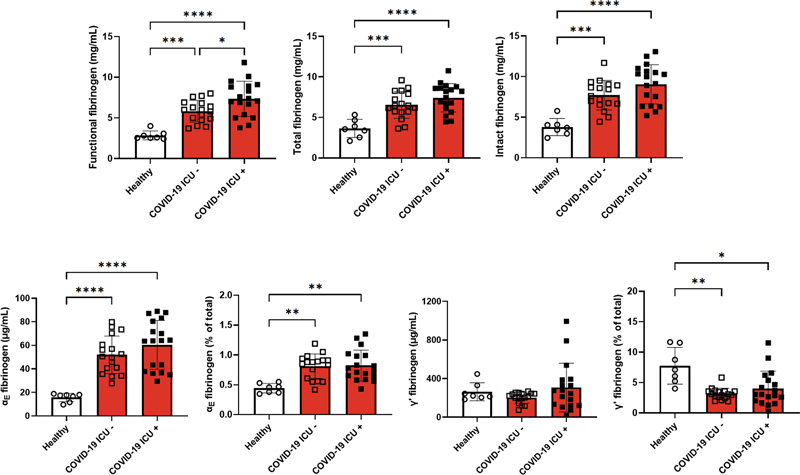
Levels of fibrinogen (variants) in plasma samples collected from healthy controls, patients with COVID-19 without thrombosis (COVID-19 ICU −), and ICU patients with COVID-19 and thrombosis after their diagnosis of thrombosis (COVID-19 +). * *p* < 0.05; ** *p* < 0.01; ** *p* < 0.001; **** *p* < 0.0001. COVID-19, coronavirus disease 2019; ICU, intensive care unit.

From ICU patients with COVID-19, plasma samples were collected at a second time point as well, namely the first available sample after the diagnosis of thrombosis (median of 11 [7–18] days since ICU admission) or at a similar time point for patients without thrombosis (median of 12 [9–15] days since ICU admission) ( Fig. 2 and Supplementary Table S1 ). In these plasma samples, we observed significantly higher functional fibrinogen, total fibrinogen, intact fibrinogen, and relative and absolute levels of α _E_ fibrinogen in both ICU patients with COVID-19 with and without thrombosis than in the healthy controls. Absolute levels of γˊ fibrinogen were similar among the groups. The decrease in relative levels of γˊ fibrinogen was more pronounced in the samples taken on the second time point and now reached significance in all ICU patients with COVID-19 (with or without thrombosis) compared to healthy controls. No differences were observed in the absolute or relative levels of fibrinogen variants between ICU patients with COVID-19 with and without thrombosis, except for a small significant difference in functional fibrinogen levels.

**Fig. 3:**
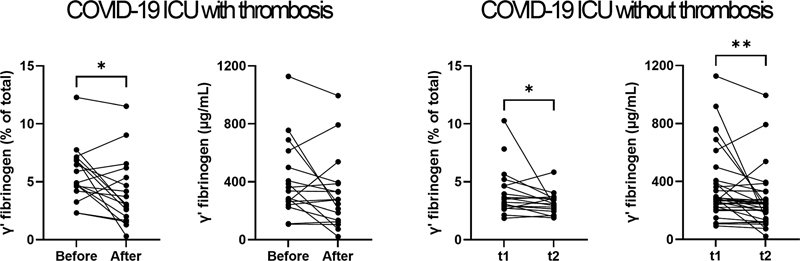
Comparison of the levels of γˊ fibrinogen in ICU patients with COVID-19 with and without thrombosis at two time points. * *p* < 0.05, ** *p* < 0.01. COVID-19, coronavirus disease 2019; ICU, intensive care unit.

The relative levels of γˊ fibrinogen significantly decreased in both ICU patients with COVID-19 with and without thrombosis between the first and second time point ( Fig. 3 ), while levels of functional, total, intact, and α _E_ fibrinogen did not change (data not shown). The decrease in the relative level of γˊ fibrinogen was not correlated with the number of days between the two plasma samples (data not shown).

### Correlations of Fibrinogen Variant Levels with Other Factors, Fibrin Network Structure, and Fibrinolysis

Functional fibrinogen levels correlated strongly with antigen levels of total and intact fibrinogen ( Supplementary Table S2 ). These fibrinogen levels showed correlations with C-reactive protein, interleukin-6, procalcitonin, plasminogen activator inhibitor 1, FVIII, FXIII, fibrin network density, turbidity change, and clot lysis time. The relative levels of α _E_ fibrinogen were positively correlated with Clauss and intact fibrinogen levels, while the relative levels of γˊ fibrinogen were not correlated to fibrinogen levels. The relative levels of α _E_ fibrinogen showed weak correlations with the turbidity change and clot lysis time, while the relative levels of γˊ fibrinogen were weakly correlated with fiber diameter.

## Discussion

Besides strongly elevated absolute levels of functional, total, and intact fibrinogen in ICU patients with COVID-19, we also showed that ICU patients with COVID-19 had significantly increased absolute and relative levels of α _E_ fibrinogen compared to healthy controls. Furthermore, fibrinogen (variant) levels were similar in ICU patients with pneumococcal infection and ICU patients with COVID-19, suggesting these increases in fibrinogen (variant) levels may be a more general observation in severe disease. Between ICU patients with COVID-19 with and without thrombosis, we did not observe differences in levels of α _E_ fibrinogen and γˊ fibrinogen, but we did observe a small significant difference in the functional fibrinogen level. Finally, the relative levels of α _E_ fibrinogen and γˊ fibrinogen were only weakly associated with fibrin network characteristics. To our knowledge, no other studies exist that measured α _E_ fibrinogen levels in patients. It has only been shown that the percentage of α _E_ fibrinogen is around 3.3% in newborns, which is higher than the 1 to 2% found in adults assessed by quantitative western blot. 31 The mechanism of the increased relative levels of α _E_ fibrinogen in ICU patients with COVID-19 remains speculative. It may be increased synthesis due to an altered alternative mRNA splicing in severe COVID-19. In addition, α _E_ fibrinogen is suggested to be less susceptible to proteolytic degradation than the normal Aα chain, possibly leading to increased relative levels in situations with upregulated synthesis of fibrinogen. 32 Finally, since we did not see a difference in relative and absolute levels of α _E_ fibrinogen between patients with and without thrombosis, we hypothesize that there is no causal relation between α _E_ fibrinogen and the risk of thrombosis.

Previous studies have shown increased relative levels of γˊ fibrinogen in patients during the acute phase of ischemic stroke. 25 Farrell et al reported high absolute levels of γˊ fibrinogen in patients with COVID-19, but did not report relative levels. 33 We initially hypothesized that severe COVID-19 would also lead to higher relative levels of γˊ fibrinogen, possibly due to severe inflammation. However, we saw decreased relative levels of γˊ fibrinogen, no correlation between inflammatory markers and relative levels of γˊ fibrinogen, and no difference in absolute levels of γˊ fibrinogen between the different groups. The mechanism explaining the decreased relative levels of γˊ fibrinogen in ICU patients with COVID-19 is unknown. It is hypothesized that alternative mRNA splicing resulting in γˊ fibrinogen occurs when an alternative polyadenylation site within the gene is used. 34 35 Previous studies have suggested that viral proteins in influenza can promote or interfere with polyadenylation. 36 This observation leads to the hypothesis that proteins of SARS-CoV-2 can possibly affect the process of polyadenylation in the fibrinogen genes and thereby reduce the relative level of γˊ fibrinogen. Furthermore, it is possible that there is increased consumption of γˊ fibrinogen in SARS-CoV-2 infection, for example, due to binding of γˊ fibrinogen to viral proteins or proteins involved in inflammatory responses. 37 Interestingly, α _E_ fibrinogen and γˊ fibrinogen did not correlate well in the current study. This observation suggests different mechanisms regulating the occurrence or stability of both mRNA splice variants and that these are differently affected by severe disease.

Interestingly, the relative and absolute levels of γˊ fibrinogen significantly decreased from the first to the second time point in ICU patients with COVID-19. The decrease in the relative levels occurred both in patients who did or did not develop thrombosis. Therefore, it is unlikely to be caused by the development of thrombosis.

Contradictory to the apparent effects of the fibrinogen variants on fibrin network structure seen in previous studies, 13 19 20 21 relative levels of the mRNA splice variants in our study were only weakly correlated with fibrin network characteristics. Previously, purified fibrinogen variants were studied instead of plasma samples. Plasma from the patients in the current study showed large variations in other (coagulation) factors, which can influence fibrin network characteristics and may explain why the association in our study is quite weak. The current correlations need confirmation in larger and/or other patient groups. Together with the finding that relative and absolute levels of α _E_ fibrinogen and γˊ fibrinogen were not significantly different between ICU patients with COVID-19 with and without thrombosis, these results suggest that the development of thrombosis in patients with COVID-19 cannot be explained by altered levels of α _E_ and γˊ fibrinogen. Also, the observation that ICU patients with pneumococcal infection showed similar fibrinogen (variant) levels to ICU patients with COVID-19 suggests that these levels cannot explain the increased development of thrombosis in severe COVID-19.

The higher functional fibrinogen levels as measured using the Clauss assay in ICU patients with COVID-19 and thrombosis compared to ICU patients with COVID-19 without thrombosis were only seen after the diagnosis of thrombosis and not at the first time point. In addition, no change in antigen levels of (total) fibrinogen was found between these two groups using the ELISAs. This points to the possibility that other coagulation factors than fibrinogen are increased or more active, resulting in higher results in the Clauss assay, and potentially contributing to the development of thrombosis.

Finally, we were interested in fibrinogen variants caused by the degradation of the α-chain in the circulating blood. This degradation results in LMW or LMWˊ fibrinogen. Currently, it is not clear what causes this degradation and which enzymes are responsible. 11 Our study shows very similar patterns for intact and total fibrinogen in the different groups, suggesting the degree of degradation of the α chain is not altered in ICU patients with COVID-19 or pneumococcal infection.

Our study has some limitations. The ICU patients with pneumococcal infection had a bacterial instead of a viral infection. Still, this control group was homogenous and showed similar symptoms to patients with COVID-19. Therefore, we considered this as our best available control group. Another potentially important difference between the groups is medication use. Anticoagulation therapy and anti-inflammatory drugs were for example differently used in the different groups, and even within the patients with COVID-19 due to changes in treatment strategies. Therefore, these differences could have affected levels of fibrinogen (variants). In addition, even though there was no clinical suspicion of thrombosis in the ICU patients with pneumococcal infection, we cannot entirely exclude the possibility that undetected thrombosis might have developed. Furthermore, the small sample sizes are a limitation. It is possible that stronger associations or differences can be observed in larger samples, which would also make it possible to adjust for covariates in the analysis. We classified ICU patients with COVID-19 into two groups based on the diagnosis of thrombosis upon imaging. However, it is the question whether this classification is really possible. It might be that all ICU patients with COVID-19 will eventually develop microthrombi that are not always detected. Finally, patients from the first and second COVID-19 waves were used, so the question remains whether these results can be generalized to patients with different viral variants.

## Conclusion

Our results show that severe COVID-19 is associated with increased levels of functional, total, intact, and α _E_ fibrinogen and decreased relative levels of γˊ fibrinogen, which may be a cause or consequence of severe disease. Since we only find a difference in functional fibrinogen and not in fibrinogen variant levels between ICU patients with COVID-19 with and without thrombosis, alterations in levels of fibrinogen variants cannot explain or predict the development of thrombosis.

## References

[1] Klok F A Kruip M JHA van der Meer N JM Incidence of thrombotic complications in critically ill ICU patients with COVID-19 Thromb Res 2020 191 145 147 32291094 10.1016/j.thromres.2020.04.013 PMC7146714

[2] Bridge K I Philippou H Ariëns R Clot properties and cardiovascular disease Thromb Haemost 2014 112 05 901 908 24899357 10.1160/TH14-02-0184

[3] Weisel J W Litvinov R I Fibrin formation, structure and properties Subcell Biochem 2017 82 405 456 28101869 10.1007/978-3-319-49674-0_13 PMC5536120

[4] Doolittle R F Fibrinogen and fibrin Annu Rev Biochem 1984 53 195 229 6383194 10.1146/annurev.bi.53.070184.001211

[5] de Maat M P Verschuur M Fibrinogen heterogeneity: inherited and noninherited Curr Opin Hematol 2005 12 05 377 383 16093783 10.1097/01.moh.0000169287.51594.3b

[6] de Vries J J Snoek C JM Rijken D C de Maat M PM Effects of post-translational modifications of fibrinogen on clot formation, clot structure, and fibrinolysis: a systematic review Arterioscler Thromb Vasc Biol 2020 40 03 554 569 31914791 10.1161/ATVBAHA.119.313626 PMC7043730

[7] Holm B Nilsen D W Kierulf P Godal H C Purification and characterization of 3 fibrinogens with different molecular weights obtained from normal human plasma Thromb Res 1985 37 01 165 176 3983897 10.1016/0049-3848(85)90043-x

[8] Holm B Brosstad F Kierulf P Godal H C Polymerization properties of two normally circulating fibrinogens, HMW and LMW. Evidence that the COOH-terminal end of the a-chain is of importance for fibrin polymerization Thromb Res 1985 39 05 595 606 4082102 10.1016/0049-3848(85)90239-7

[9] Gorkun O V Veklich Y I Medved L V Henschen A H Weisel J W Role of the alpha C domains of fibrin in clot formation Biochemistry 1994 33 22 6986 6997 8204632 10.1021/bi00188a031

[10] Hasegawa N Sasaki S Location of the binding site “b” for lateral polymerization of fibrin Thromb Res 1990 57 02 183 195 2315883 10.1016/0049-3848(90)90318-7

[11] Kaijzel E L Koolwijk P van Erck M G van Hinsbergh V W de Maat M P Molecular weight fibrinogen variants determine angiogenesis rate in a fibrin matrix in vitro and in vivo J Thromb Haemost 2006 4 09 1975 1981 16961604 10.1111/j.1538-7836.2006.02081.x

[12] Fu Y Weissbach L Plant P W Carboxy-terminal-extended variant of the human fibrinogen alpha subunit: a novel exon conferring marked homology to beta and gamma subunits Biochemistry 1992 31 48 11968 11972 1457396 10.1021/bi00163a002

[13] Mosesson M W DiOrio J P Hernandez I Hainfeld J F Wall J S Grieninger G The ultrastructure of fibrinogen-420 and the fibrin-420 clot Biophys Chem 2004 112(2-3):209 214 15572250 10.1016/j.bpc.2004.07.021

[14] Mosesson M W Fibrinogen gamma chain functions J Thromb Haemost 2003 1 02 231 238 12871494 10.1046/j.1538-7836.2003.00063.x

[15] Baker S R Ariëns R AS Chapter 3 - Fibrin clot structure and function: a novel risk factor for arterial and venous thrombosis and thromboembolism Cambridge, MA Academic Press 2018 31 49

[16] Allan P Uitte de Willige S Abou-Saleh R H Connell S D Ariëns R A Evidence that fibrinogen γ′ directly interferes with protofibril growth: implications for fibrin structure and clot stiffness J Thromb Haemost 2012 10 06 1072 1080 22463367 10.1111/j.1538-7836.2012.04717.x

[17] Uitte de Willige S Standeven K F Philippou H Ariëns R A The pleiotropic role of the fibrinogen gamma' chain in hemostasis Blood 2009 114 19 3994 4001 19687509 10.1182/blood-2009-05-217968

[18] Farrell D H γ′ Fibrinogen as a novel marker of thrombotic disease Clin Chem Lab Med 2012 50 11 1903 1909 23091268 10.1515/cclm-2012-0005

[19] Cooper A V Standeven K F Ariëns R A Fibrinogen gamma-chain splice variant gamma' alters fibrin formation and structure Blood 2003 102 02 535 540 12663453 10.1182/blood-2002-10-3150

[20] Siebenlist K R Mosesson M W Hernandez I Studies on the basis for the properties of fibrin produced from fibrinogen-containing gamma' chains Blood 2005 106 08 2730 2736 16002430 10.1182/blood-2005-01-0240 PMC1895298

[21] Gersh K C Nagaswami C Weisel J W Lord S T The presence of gamma' chain impairs fibrin polymerization Thromb Res 2009 124 03 356 363 19138790 10.1016/j.thromres.2008.11.016 PMC2752440

[22] Lovely R S Kazmierczak S C Massaro J M D'Agostino R B Sr O'Donnell C J Farrell D H Gamma' fibrinogen: evaluation of a new assay for study of associations with cardiovascular disease Clin Chem 2010 56 05 781 788 20348406 10.1373/clinchem.2009.138347 PMC3033762

[23] Lovely R S Falls L A Al-Mondhiry H A Association of gammaA/gamma' fibrinogen levels and coronary artery disease Thromb Haemost 2002 88 01 26 31 12152671

[24] Mannila M N Lovely R S Kazmierczak S C Elevated plasma fibrinogen gamma' concentration is associated with myocardial infarction: effects of variation in fibrinogen genes and environmental factors J Thromb Haemost 2007 5 04 766 773 17263791 10.1111/j.1538-7836.2007.02406.x

[25] Cheung E Y Uitte de Willige S Vos H L Fibrinogen gamma' in ischemic stroke: a case-control study Stroke 2008 39 03 1033 1035 18239174 10.1161/STROKEAHA.107.495499

[26] Pronto-Laborinho A C Lopes C S Conceição V A γ′ Fibrinogen as a predictor of survival in amyotrophic lateral sclerosis Front Cardiovasc Med 2021 8 715842 34568457 10.3389/fcvm.2021.715842 PMC8458885

[27] Uitte de Willige S de Visser M C Houwing-Duistermaat J J Rosendaal F R Vos H L Bertina R M Genetic variation in the fibrinogen gamma gene increases the risk for deep venous thrombosis by reducing plasma fibrinogen gamma' levels Blood 2005 106 13 4176 4183 16144795 10.1182/blood-2005-05-2180

[28] Dutch COVID Thrombosis Coalition study group Kruip M JHA Cannegieter S C Ten Cate H Caging the dragon: research approach to COVID-19-related thrombosis Res Pract Thromb Haemost 2021 5 02 278 290 33733026 10.1002/rth2.12470 PMC7938618

[29] de Vries J J Visser C Geers L Altered fibrin network structure and fibrinolysis in intensive care unit patients with COVID-19, not entirely explaining the increased risk of thrombosis J Thromb Haemost 2022 20 06 1412 1420 35316570 10.1111/jth.15708 PMC9115158

[30] de Maat M P van Schie M Kluft C Leebeek F W Meijer P Biological variation of hemostasis variables in thrombosis and bleeding: consequences for performance specifications Clin Chem 2016 62 12 1639 1646 27679435 10.1373/clinchem.2016.261248

[31] Grieninger G Lu X Cao Y Fib420, the novel fibrinogen subclass: newborn levels are higher than adult Blood 1997 90 07 2609 2614 9326227

[32] Fu Y Grieninger G Fib420: a normal human variant of fibrinogen with two extended alpha chains Proc Natl Acad Sci U S A 1994 91 07 2625 2628 8146165 10.1073/pnas.91.7.2625 PMC43422

[33] Farrell D H Hudkins M Hamilton H Abstract 9308: extreme gamma prime fibrinogen levels in COVID-19 patients Circulation 2021 144 A9308 A9308

[34] Fornace A J Jr Cummings D E Comeau C M Kant J A Crabtree G R Structure of the human gamma-fibrinogen gene. Alternate mRNA splicing near the 3′ end of the gene produces gamma A and gamma B forms of gamma-fibrinogen J Biol Chem 1984 259 20 12826 12830 6092346

[35] Chung D W Davie E W gamma and gamma' chains of human fibrinogen are produced by alternative mRNA processing Biochemistry 1984 23 18 4232 4236 6091742 10.1021/bi00313a033

[36] Lutz C S Alternative polyadenylation: a twist on mRNA 3′ end formation ACS Chem Biol 2008 3 10 609 617 18817380 10.1021/cb800138w

[37] Sangith N Unique fibrinogen-binding motifs in the nucleocapsid phosphoprotein of SARS CoV-2: Potential implications in host-pathogen interactions Med Hypotheses 2020 144 110030 32758876 10.1016/j.mehy.2020.110030 PMC7313483

