# Supplementary material for: Levels of Fibrinogen Variants Are Altered in Severe COVID-19
Source: TH Open. 2023 Jul 13;7(3):e217–25. doi: 10.1055/a-2102-4521 (PMC10370639; doi:10.1055/a-2102-4521)
Supplement: Supplementary file 1 — Supplementary Material [file 10-1055-a-2102-4521-s23020006.pdf]

Supplementary Table S1 Absolute and relative levels of fibrinogen (variants)

	Healthy controls ($n=7$)	Ward patients with COVID-19 ($n=10$)	ICU patients with COVID-19 without thrombosis ($n=18$)		ICU patients with COVID-19 with thrombosis ($n=19$)		ICU patients with pneumococcal infection ($n=6$)
			Time point 1	Time point 2	Before	After	
Clauss fibrinogen (mg/mL)	2.9 ± 0.5	3.6 ± 0.6	6.0 ± 1.6	5.9 ± 1.3	6.4 ± 2.5	7.3 ± 2.1	8.4 ± 2.7
Total fibrinogen (mg/mL)	3.6 ± 1.1	5.3 ± 1.0	7.1 ± 2.0	6.5 ± 1.7	6.8 ± 2.0	7.4 ± 1.7	7.4 ± 1.8
Intact fibrinogen (mg/mL)	3.8 ± 1.1	5.6 ± 1.0	8.2 ± 2.0	7.7 ± 1.8	8.2 ± 2.5	9.1 ± 2.4	9.3 ± 2.1
α_E fibrinogen (µg/mL)	15.7 ± 3.6	29.7 ± 8.7	56.2 ± 19.2	52.0 ± 15.9	56.5 ± 23.5	60.4 ± 20.7	51.0 ± 14.1
α_E fibrinogen (% of total)	0.4 ± 0.1	0.6 ± 0.2	0.8 ± 0.2	0.8 ± 0.2	0.8 ± 0.2	0.8 ± 0.3	0.8 ± 0.5
γ′ fibrinogen µg/mL)	223 [210–289]	228 [190–292]	256 [201–289]	212 [195–244]	312 [263–500]	262 [124–388]	214 [166–519]
γ′ fibrinogen (% of total)	7.1 [5.6–10.2]	4.5 [3.5–5.7]	3.6 [2.9–4.7]	3.1 [2.7–3.6]	4.8 [4.6–6.9]	3.1 [2.0–5.4]	2.5 [2.1–6.1]

Abbreviation: ICU, intensive care unit.
Note: Mean ± SD or median [25th–75th percentile].

 © 2023. The Author(s).

Supplementary Table S2 Correlations between fibrinogen variants and (coagulation) factors and fibrin network characteristics

	Clauss (mg/mL)	Total (mg/mL)	Intact (mg/mL)	α_E (µg/mL)	α_E (%)	γ´ (µg/mL)	γ´ (%)
Clauss (mg/mL)		0.86**	0.92**	0.77**	0.43**	0.39**	−0.11
Total (mg/mL)	0.86**		0.93**	0.75**	0.25	0.47**	−0.13
Intact (mg/mL)	0.92**	0.93**		0.82**	0.41**	0.43**	−0.12
α_E (µg/mL)	0.77**	0.75**	0.82**		0.80**	0.30*	−0.17
α_E (%)	0.43**	0.25	0.41**	0.80**		0.05	−0.09
γ´ (µg/mL)	0.39**	0.47**	0.43**	0.30*	0.05		0.73**
γ´ (%)	−0.11	−0.13	−0.12	−0.17	−0.09	0.73**	
D-dimer (mg/L)	0.32*	0.17	0.25	0.38**	0.41**	0.08	−0.19
Anti-Xa (U/mL)	0.23	0.21	0.30*	0.36**	0.34**	−0.07	−0.31*
C-reactive protein (mg/L)	0.77**	0.63**	0.73**	0.68**	0.39**	0.34*	0.08
Interleukin-6 (pg/mL)	0.64**	0.76**	0.70**	0.77**	0.34	0.35	0.04
Procalcitonin (ng/mL)	0.50**	0.33	0.43*	0.40*	0.18	0.44**	0.33
FVIII (U/mL)	0.27*	0.26*	0.34**	0.43**	0.37**	−0.17	−0.47**
FXIII (U/mL)	−0.39**	−0.24	−0.29*	−0.40**	−0.39**	−0.21	0.01
PAI-1 (ng/mL)	0.53**	0.46**	0.54**	0.57**	0.41**	0.09	−0.27*
Fiber diameter (nm)	0.01	−0.06	0.01	−0.02	0.03	0.19	0.31*
Fibrin network density (%)	0.23	0.33*	0.28*	0.27*	0.14	−0.03	−0.25
Turbidity change (AU)	0.75**	0.84**	0.89**	0.78**	0.44**	0.33*	−0.15
Clot lysis time (min)	0.45**	0.44**	0.49**	0.45**	0.27*	0.10	−0.20

Abbreviations: AU, arbitrary units; PAI-1, plasminogen activator inhibitor 1.
Note: Spearman correlation coefficients in samples of all individuals together at the first time point. *$p < 0.05$, **$p < 0.01$.

 © 2023. The Author(s).
